# How Does Lymphocyte Depletion Affect Rectal Cancer Radiotherapy Outcomes?

**DOI:** 10.7759/cureus.105398

**Published:** 2026-03-17

**Authors:** Zineb Dahbi, Reyzane El Mjabber, Rim Alami, Yasmine Harrar, Fadila Guessous

**Affiliations:** 1 Radiation Oncology, Mohamed VI university of sciences and health (UM6SS), Casablanca, MAR; 2 Radiation Oncology, Cheikh Khalifa International University Hospital, Casablanca, MAR; 3 Allergy and Immunology, Mohamed VI university of sciences and health (UM6SS), casablanca, MAR

**Keywords:** hematopoietic bone marrow, pelvic radiotherapy, radiation-related lymphopenia, rectal cancer, tumor micro environment

## Abstract

Background

Pelvic radiotherapy for rectal cancer inevitably exposes substantial volumes of hematopoietic bone marrow, which are highly susceptible to radiation-induced damage. This leads to lymphopenia, with implications for both immune function and tumor control. The impact of lymphocyte depletion during treatment, particularly its correlation with pathological response, local control, and overall survival, remains an area of active research.

Methods

In this study, we analyzed lymphocyte variations during radiotherapy for rectal cancer and their relationship to treatment outcomes. Delta lymphocyte count was used as a marker for radiation-induced lymphopenia (RIL), defined as the difference between the highest and lowest lymphocyte levels during treatment. A cohort of rectal cancer patients receiving pelvic radiotherapy was followed to assess the relationship between radiation-induced lymphopenia and progression-free survival, pathological response, and treatment-related toxicity, including mucositis, dermatitis, and xerostomia.

Results

A total of 97 patients were included, with a median age of 69 years. Tumors were mostly located less than 5 cm from the anal margin (53.4%). The majority had T3-T4 disease (59.7%), and 80.4% had clinically positive lymph nodes. Treatment included external radiotherapy (mean dose 50.2 Gy) and chemotherapy, with 92.8% receiving 3D radiotherapy. The median follow-up was 72 months. The incidence of grade 3-4 lymphopenia was 25.21%, with a mean delta lymphopenia (ΔL) of 350.6 cells. The Tumor Regression Grade (TRG) score showed 61.2% had a response of >1. Higher radiation doses (>25 Gy) were associated with greater reductions in lymphocyte counts (Spearman correlation = 0.45, p=0.03). Kaplan-Meier analysis revealed better progression-free survival for patients with ΔL ≤300 cells (HR=0.65, p=0.0013). Multivariate analysis confirmed that ΔL was a significant predictor of progression-free survival (HR=0.68, p=0.012), with larger ΔL values linked to poorer survival.

Conclusion

Delta lymphocyte count serves as a reliable biomarker for radiation-induced immune suppression and is predictive of pathological response and survival outcomes in rectal cancer patients undergoing pelvic radiotherapy. Our findings underscore the importance of considering immune preservation in radiotherapy planning. Tailored interventions, such as marrow-sparing techniques and immunomodulatory strategies, could potentially improve treatment efficacy and reduce toxicities.

## Introduction

The standard treatment for locally advanced rectal cancer is total neoadjuvant therapy (TNT) [1,2]. This approach consists of induction chemotherapy followed by chemoradiotherapy, as established in the PRODIGE-23 protocol [2], or short-course radiotherapy followed by systemic chemotherapy, as evaluated in the RAPIDO trial [1]. Both approaches are considered equivalent [3,4], with surgery performed when indicated.

Over the past two decades, cancer biology has shifted from a tumor-focused view to one recognizing the tumor microenvironment (TME) as a complex ecosystem of stromal and immune components that critically influence tumor progression and therapeutic response [5-8]. Growing evidence has also highlighted the TME's contribution to treatment resistance, influencing both systemic therapies and radiotherapy responses [9-11].

Lymphocytes are highly radiosensitive, and even modest radiation exposure can cause significant declines in circulating lymphocyte counts [12]. Because the immune system plays a central role in antitumor activity, the presence of CD8+ and CD4+ tumor-infiltrating lymphocytes has been associated with improved survival in esophageal cancer, owing to their capacity to directly eliminate malignant cells or to secrete cytokines that stimulate additional immune effectors [13,14]. Radiation-induced lymphopenia (RIL)-the decline in circulating lymphocytes observed during radiotherapy-has been correlated with inferior outcomes across multiple malignancies, including rectal cancer, gliomas, head and neck cancers, lung cancer, and pancreatic cancer [15-20]. These observations suggest that RIL may reflect systemic immune suppression and could serve as a prognostic indicator of tumor control and overall survival.

In patients with pelvic malignancies, including rectal cancer, radiotherapy frequently involves irradiation of large volumes of lymphoid-rich structures, such as the pelvic bone marrow, iliac vessels, and regional lymph nodes [21-24] Given that a substantial proportion of hematopoietic activity-up to one quarter-occurs in the pelvis, exposure of these sites can significantly impair circulating lymphocyte counts and overall immune function [25]. Radiation-induced lymphopenia (RIL) is a common consequence of pelvic irradiation and has been associated with worse clinical outcomes in multiple tumor types [26]. Observational studies indicate that higher radiation doses and larger irradiated volumes of pelvic bone marrow correlate with increased hematologic toxicity, although precise dose-volume thresholds remain undetermined [22,23,27,28]. In the absence of validated normal tissue complication probability (NTCP) models, the ALARA principle ("as low as reasonably achievable") has been recommended to minimize lymphocyte depletion during pelvic radiotherapy [21]. Collectively, these findings suggest that preserving lymphocyte counts may be crucial for optimizing tumor control and survival in rectal cancer patients undergoing radiotherapy; for this reason, we selected lymphopenia as the primary marker of the immune response.

Delta lymphocyte count was selected as the primary biomarker of radiation-induced lymphopenia because it provides a dynamic assessment of immune depletion during treatment, integrating both baseline variability and treatment-related lymphocyte loss, which may better correlate with clinical outcomes than isolated baseline or nadir values.

Aims

The primary objective of this study was to evaluate the association between lymphocyte dynamics during radiotherapy and progression-free survival (PFS). Secondary objectives included analysis of pathological response (Tumor Regression Grade; TRG), while local control and overall survival were explored descriptively.

## Materials and methods

Study design and population

We conducted a retrospective review of patients with histologically confirmed rectal adenocarcinoma treated at a single institution with neoadjuvant pelvic radiotherapy between January 2016 and January 2024. Patients managed within a total neoadjuvant treatment strategy were eligible for inclusion. Exclusion criteria comprised the presence of metastatic disease at diagnosis, prior pelvic irradiation, synchronous malignancies, or pre-existing hematological disorders. None of the included patients required granulocyte-monocyte colony-stimulating factor support or received red blood cell or platelet transfusions during the course of pelvic radiotherapy. Variations in lymphocyte counts were graded according to the Common Terminology Criteria for Adverse Events (CTCAE), version 5.0 [29,30].

Study protocol and data collection

For each patient, clinical and demographic data were collected from electronic medical records using the DX Care system, including age, sex, tumor histological subtype, tumor location, circumferential resection margin status, and TNM staging [31]. Details regarding neoadjuvant chemotherapy and radiotherapy protocols were recorded, along with oncologic outcomes. Postoperative pathological reports were reviewed to evaluate tumor response using the Dworak Tumor Regression Grading (TRG) system, which classifies response from grade 0 (no regression) to grade 4 (complete regression) [29].

Radiotherapy dosimetric assessment

Radiotherapy target volumes were defined according to institutional practice and international guidelines [32]. The gross tumor volume (GTV) included the primary rectal tumor and involved lymph nodes. The clinical target volume (CTV) encompassed the mesorectum and elective pelvic nodal regions when indicated. A planning target volume (PTV) margin of 5-10 mm was applied to account for setup uncertainty and organ motion. Treatments were delivered using three-dimensional conformal radiotherapy or intensity-modulated radiotherapy with megavoltage photon beams (6-18 MV), using CT-based dose calculation and daily or weekly image guidance.

Pelvic bone marrow was contoured on simulation CT scans and included the ilium, sacrum, ischium, and proximal femora, extending from the L5-S1 junction to the inferior margin of the ischial tuberosities. Based on established radioanatomical guidelines, two distinct bone marrow compartments were delineated. Low-density bone marrow (LDBM) was identified as hypodense regions within pelvic bones on simulation CT, while active bone marrow (ABM) corresponded to relatively hyperdense trabecular marrow areas, following previously described CT-based marrow delineation approaches. Contouring was performed using anatomical landmarks by experienced radiation oncologists. No strict Hounsfield unit thresholds or interobserver variability assessments were applied due to the retrospective nature of the study.

No modifications were made to the original pelvic radiotherapy treatment plans. Dosimetric parameters, including mean dose, maximum dose, and dose-volume metrics (V10, V30, and V40) for pelvic bone marrow, were extracted from the treatment planning system.

The biologically effective dose (BED) was calculated using the linear-quadratic model with an α/β ratio of 10 Gy, appropriate for rectal adenocarcinoma. A mean pelvic bone marrow dose threshold of >25 Gy was predefined for exploratory correlation analyses with delta lymphocyte count.

Biological data collection

Peripheral lymphocyte counts were obtained at baseline, during radiotherapy as part of routine clinical monitoring, and at follow-up visits at three and six months after treatment. Delta-lymphocyte variation (ΔL) was defined as the difference between the highest and lowest recorded lymphocyte values across these time points.

Statistical analysis

Statistical analyses were performed using SPSS software, version 17.0 (IBM Inc., Armonk, New York). Paired comparisons were conducted using the paired-sample t-test. A two-sided p-value of <0.05 was considered statistically significant. Correlations between variables were assessed using Spearman’s rank correlation coefficient. Survival outcomes were estimated using the Kaplan-Meier method, and differences between groups were evaluated using the log-rank test. Multivariable Cox regression models were adjusted for relevant clinical variables, including age, tumor stage, and mean pelvic bone marrow dose. 

## Results

A total of 97 patients were included in the analysis. The median age was 69 years. 54.3% were male patients. More than half of the tumors (53.4%) were located less than 5 cm from the anal margin. 62.8% of patients had tumors located within 1 mm from the anal margin. The clinical T stage distribution was as follows: T1 (13.4%), T2 (26.8%), 59.7% of the patients had T3-T4 disease. Clinically positive lymph nodes were observed in 80.4% of patients (Table 1).

**Table 1:** Baseline clinical and tumor characteristics of the study population MRF - mesorectal fascia

Parameter	Subcategory / value	N (%)
Age	Mean (Years)	69 (100%)
			Sex	Male	54 (54.28%)
Female	43 (45.72%)		
Distance from anal margin (cm)	<5 cm	52 (53.44%)
	5-10 cm	48 (46.56%)
			Distance to MRF	<1mm	61 (62.8%)
Clinical T stage	T1	13 (13.4%)
	T2	26 (26.8%)
	T3	32 (32.9%)
	T4	26 (26.8%)
Clinical N stage	N0	19 (19.55%)
	N+	78 (80.4%)

The treatments administered to the patients included external radiotherapy, with a mean total dose of 50.2 Gy, ranging from 25 Gy to 66 Gy, and a mean dose per fraction of 1.9 Gy, varying from 1.8 Gy to 5 Gy. The radiotherapy techniques used were predominantly 3D (92.8%), while intensity-modulated radiation therapy (IMRT) was used in 7.2% of cases. Regarding specific neoadjuvant radiotherapy protocols, 35% (34 patients) were treated with the PRODIGE 23 protocol, 15% (15 patients) followed the RAPIDO protocol, and 49% (48 patients) received other neoadjuvant radiotherapy treatments. These patients received neoadjuvant regimens with institution-specific chemotherapy schedules administered either before or after radiotherapy, according to multidisciplinary team decisions. The mean duration of radiotherapy treatment was 39 days, and the median follow-up period was 72 months.

The median volumes of LDBM and ABM were 114.6 cc and 765.4 cc, respectively. The maximal doses received by LDBM and ABM were 54.2 Gy and 52.2 Gy, respectively. Mean dose to BM wasn't statistically different if considering contouring BM based on LDBM or ABM (29.3 Gy versus 30.8 Gy, p=0.64)

The incidence of grade 3-4 lymphopenia was 25.21%, with a mean delta lymphopenia (ΔL) of 350,6 elements. No grade V toxicity was reported in our study. Histologic response was evaluated using the TRG score, where >1 was 61.2% (Table 2).

**Table 2:** Comparison of dosimetric parameters between low-density and active bone marrow volumes Dmax - maximal dose; Dmean - mean dose; V10 - volume in cc receiving 10 Gy during the whole treatment; V40 - volume in cc receiving 40 Gy during the whole treatment; LDBM -low-density bone marrow; ABM - active bone marrow Paired sample T test was used for statistical analysis; p<0.05 was considered significant

Parameter	LDBM	ABM	T	p-value
Volume cc	1144.6	765.3	1.33	0.034
Dmax	54.2	52.1	0.34	0.12
Dmean	29.3	30.7	0.29	0.64
V10 Gy	881.2	664.6	4.55	0.0145
V40 Gy	410.8	36.43	3.51	0.001

Spearman's correlation between (Mean dose BM >25 Gy) and delta lymphopenia (>300 E) was 0.45 (p=0.03), indicating higher radiation doses were associated with greater reductions in lymphocyte counts.

**Figure 1:**
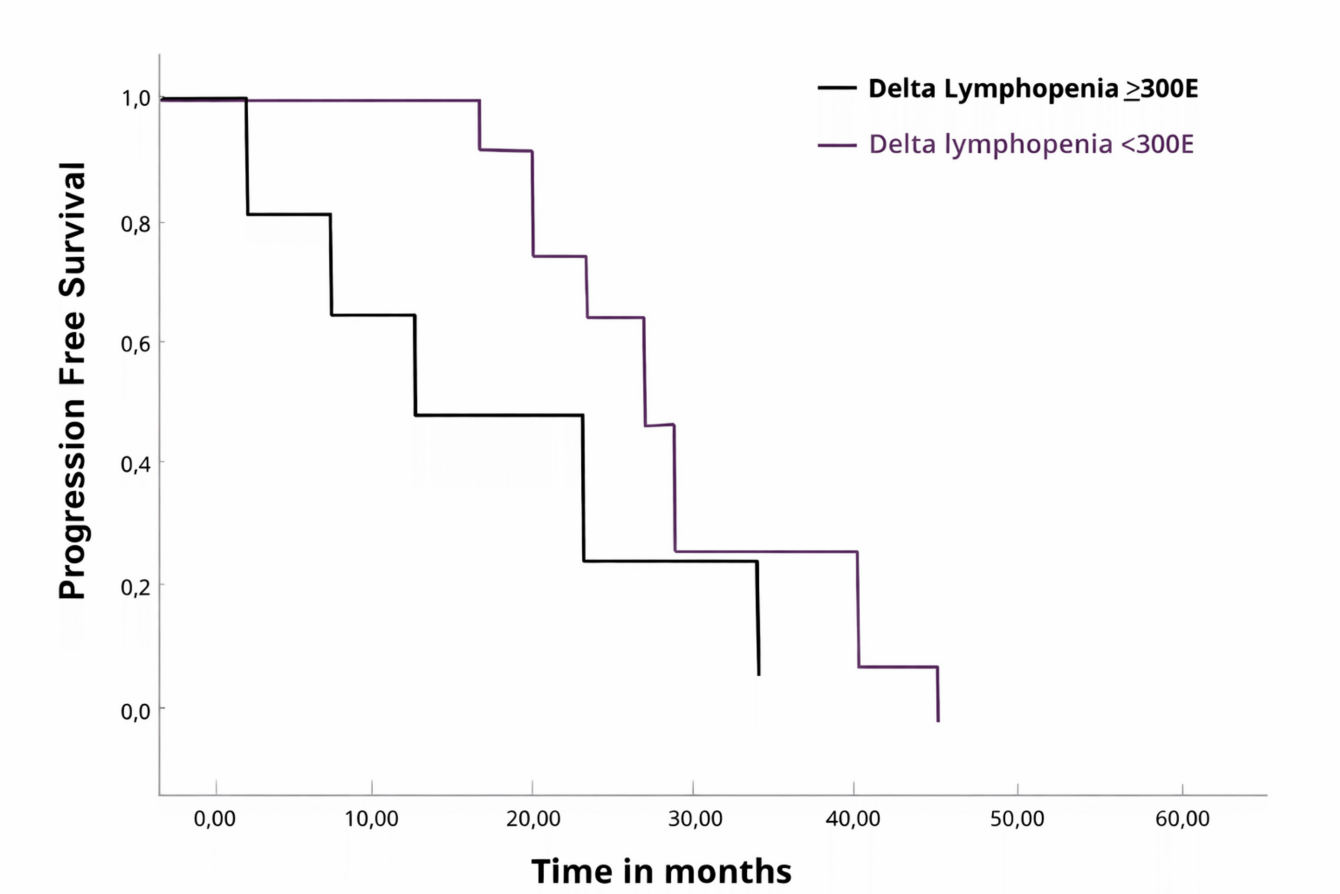
Impact of Δ-lymphopenia on progression-free survival / analysis of progression-free survival in patients according to variations in lymphocyte levels (Δ lymphopenia) observed throughout the treatment course, highlighting the impact of immune status on disease control

The Kaplan-Meier analysis showed that lymphocytes variation (ΔL) less than or equal to 300E had significantly better progression-free survival (HR=0.65, p=0.0013) (Figure 1).

The multivariate analysis revealed that ΔL remained a significant predictor of PFS after adjusting for other factors (hazard ratio (HR)=0.68, 95% confidence interval (CI): 0.50-0.92, p=0.012). In contrast, age and tumor stage were not found to be statistically significant predictors of PFS in this cohort. Specifically, while higher ΔLvalues (>300E) were associated with poorer survival, this effect was adjusted for the influence of other covariates in the model (Table 3).

**Table 3:** Univariate and multivariate analysis of factors associated with tumor regression grade (TRG) in rectal cancer patients

Variables	Univariate analysis		Multivariate analysis	
Cox regression	HR-95%IC	p-valuie	HR-95%IC	p-value
Age	1.2 (1.84-1.99)	0.112	1.33 (1.74-1.89)	0.245
Histological subtype	0.65 (0.57-0.77)	0.276	0.25 (0.11-0.57)	0.375
Tumor stage T	1.67 (1.98-1.34)	0.18	1.55 (1.47-1.79)	0.27
Delta-lymphocytes	1.50(1.20-1.80)	0.003	1.45 (0.79-1.67)	0.004
Mean dose BM	1.3- (1.28-1.92)	0.001	1.56 (0.28-1.92)	0.002
TRG score >1	2.1 (1.5-2.9)	0.0001	2.05 (0.45-2.8)	0.001

## Discussion

Pelvic radiotherapy in rectal cancer unavoidably delivers radiation to large volumes of active bone marrow, most notably within the iliac, sacral, and ischial regions. As the pelvis harbors a substantial share of the body's hematopoietically functional marrow, these anatomical sites are particularly prone to radiation-related hematologic toxicity [33,34]. Radiation exposure affects immune homeostasis through two complementary mechanisms: direct depletion of circulating lymphocytes, which exhibit high radiosensitivity, and disruption of hematopoietic stem cells and lymphoid progenitors responsible for cellular renewal. The combination of these effects may result in marked and persistent lymphopenia, a condition that has been associated with impaired tumor control and decreased survival [12,17]. From a radiobiological standpoint, these observations illustrate how tissue-specific radiosensitivity and the spatial distribution of stem cell populations influence the biological consequences of radiation dose, thereby emphasizing the clinical relevance of Δ-lymphocyte variation, which may serve as a potential clinical marker reflecting radiation-induced lymphopenia and its association with treatment outcomes [35-37]. Identifying patients with large lymphocyte drops may enable tailored interventions, such as marrow-sparing radiotherapy planning, dose constraints on low- and medium-dose pelvic volumes, or incorporation of immunomodulatory strategies. In conclusion, the magnitude of radiation-induced lymphopenia, as captured by delta lymphocyte count, is independently associated with pathological response, local tumor control, and overall survival in rectal cancer patients undergoing pelvic radiotherapy [38-42].

Limitations

This study has several limitations. Its retrospective, single-center design may introduce selection bias and limit the generalizability of the findings. The sample size, although comparable to other studies evaluating radiation-induced lymphopenia in rectal cancer, may have limited the statistical power to detect weaker associations in multivariate analyses.

Treatment heterogeneity represents another important limitation. Patients received different neoadjuvant strategies, radiotherapy doses, fractionation schemes, and chemotherapy regimens, which may have influenced lymphocyte dynamics and clinical outcomes. In addition, most patients were treated using three-dimensional conformal radiotherapy, with limited use of contemporary bone marrow-sparing techniques; therefore, the extent of lymphocyte depletion observed in this cohort may not fully reflect outcomes achievable with modern IMRT or volumetric modulated arc therapy (VMAT) approaches.

Although lymphocyte counts were routinely monitored on a weekly basis during radiotherapy, variability in sampling frequency and timing may have influenced the calculation of delta lymphocyte count (ΔL), introducing potential measurement bias. Moreover, ΔL represents a composite biomarker affected by multiple factors beyond radiotherapy, including baseline immune status, chemotherapy exposure, infections, corticosteroid use, and post-treatment immune recovery. Consequently, exclusive attribution of lymphocyte depletion to radiation exposure is not possible in this retrospective analysis.

Immune assessment was limited to total peripheral lymphocyte counts, without evaluation of lymphocyte subpopulations or functional immune markers, which restricts biological interpretation. Bone marrow segmentation was based on qualitative CT appearance without predefined Hounsfield unit thresholds or interobserver variability assessment, potentially limiting reproducibility and introducing observer-related variability.

Finally, while progression-free survival was the primary endpoint of this study, analyses of local control and overall survival were limited by the number of events and should be interpreted with caution. The ΔL cutoff value used in this study was exploratory and requires validation in prospective, multicenter cohorts.

Despite these limitations, this study provides clinically relevant data supporting the prognostic significance of lymphocyte dynamics during pelvic radiotherapy for rectal cancer and highlights the need for immune-preserving radiotherapy strategies in future investigations.

## Conclusions

Radiotherapy-induced lymphopenia is a frequent and clinically significant phenomenon in patients with rectal cancer undergoing pelvic irradiation. Our study suggests that greater reductions in circulating lymphocyte counts (Δlymphocytes >300 cells/µL) are associated with poorer progression-free survival and lower rates of complete pathological response, highlighting the prognostic value of immune monitoring during treatment. Dosimetric factors, particularly the mean dose to pelvic bone marrow, are strongly correlated with lymphocyte depletion, emphasizing the need to incorporate bone marrow-sparing strategies into radiotherapy planning. These findings illustrate the dual impact of radiotherapy on the immune system: while it contributes to local tumor control, excessive irradiation of hematopoietic compartments may compromise systemic immunity and overall treatment efficacy. Future prospective studies integrating immune-preserving radiotherapy techniques, along with combinations of radiotherapy and immunotherapy, are warranted to optimize both local control and systemic anti-tumor immunity in rectal cancer.
